# Poster Session I - A113 PRACTICES AND PERCEPTIONS OF ADVANCED ENDOSCOPIC IMAGING TECHNIQUES FOR COLORECTAL NEOPLASIA DETECTION IN PATIENTS WITH INFLAMMATORY BOWEL DISEASE (IBD): A SURVEY AMONG CANADIAN IBD SPECIALISTS

**DOI:** 10.1093/jcag/gwaf042.113

**Published:** 2026-02-13

**Authors:** R Dang, L M van Lierop, T Bessissow, N Narula, T Ramsay, P Greenstreet, F Hoentjen, S Murthy

**Affiliations:** McMaster University, Hamilton, ON, Canada; Gastroenterology, University of Alberta Faculty of Medicine & Dentistry, Edmonton, AB, Canada; McGill University, Montreal, QC, Canada; McMaster University, Hamilton, ON, Canada; Ottawa Hospital Research Institute Ottawa Methods Centre, Ottawa, ON, Canada; Ottawa Hospital Research Institute Ottawa Methods Centre, Ottawa, ON, Canada; Gastroenterology, University of Alberta Faculty of Medicine & Dentistry, Edmonton, AB, Canada; University of Ottawa, Ottawa, ON, Canada

## Abstract

**Background:**

Dye chromoendoscopy (DCE) and virtual chromoendoscopy (VCE) are recommended as first-line adjuncts in conjunction with high-definition white light endoscopy (HD-WLE) for colorectal neoplasm (CRN) detection in inflammatory bowel disease (IBD).

**Aims:**

This study surveyed Canadian IBD specialists to understand their perceptions, preferences, and practices regarding image-enhancement modalities (including DCE, VCE, and Artificial Intelligence (AI)-aided endoscopy) for CRN detection in IBD, to inform future studies and clinical recommendations.

**Methods:**

Developed collaboratively by IBD experts and methodologists, the survey assessed physicians’ attitudes toward colonoscopy image-enhancement modalities for CRN surveillance in IBD, evaluating five domains: current practices, familiarity with advanced techniques, perceptions of AI-assisted endoscopy, barriers to adoption, and research priorities. It was distributed to IBD specialists affiliated with the Canadian IBD Research Consortium and conducted per CROSS guidelines.

**Results:**

Of 82 specialists invited, 33 responded (40.2%). Most worked in academic centers (90%); 39.4% managed large IBD practices. VCE was the most used advanced imaging modality (84.8%), followed by DCE (33.3%), HD-WLE plus non-targeted biopsies (33.3%), AI-assisted colonoscopy (18.2%), and HD-WLE alone (6.1%). Although 60.6% considered DCE and VCE similarly effective, drawbacks included need for meticulous bowel preparation (VCE 26.6%, DCE 24.1%) and poor visualization on a background of active inflammation (VCE 26.6%, DCE 12%). AI-assisted colonoscopy was seen as promising for CRN detection; 78.8% agreed randomized controlled trials are needed, and 39.4% would adopt AI if it improved CRN detection by 5% over HD-WLE.

**Conclusions:**

This survey highlights a shift towards VCE over DCE, perceived drawbacks of chromoendoscopy, limited availability and uncertainty regarding AI-assisted colonoscopy, and a desire for future research to demonstrate AI’s utility for CRN detection in IBD.

**Funding Agencies:**

None

